# Telehealth Adoption Among Saudi Older Adults: A Qualitative Analysis of Utilization and Barriers

**DOI:** 10.3390/healthcare12232470

**Published:** 2024-12-06

**Authors:** Abdulaziz M. Alodhialah, Ashwaq A. Almutairi, Mohammed Almutairi

**Affiliations:** 1 Department of Medical Surgical Nursing, College of Nursing, King Saud University, Riyadh 11451, Saudi Arabia; mohalmutairi@ksu.edu.sa; 2 School of Nursing & Midwifery, Monash University, Melbourne, VIC 3004, Australia; ashwaq.almutairi@monash.edu

**Keywords:** telehealth, older adults, digital literacy, healthcare access, Saudi Arabia

## Abstract

Background/Objectives: The rapid adoption of telehealth services has been significantly accelerated by the need for accessible healthcare solutions, especially among older adults. However, the utilization of telehealth remains limited in many regions, including Saudi Arabia. This study aims to identify the barriers and facilitators influencing telehealth adoption among older adults in Riyadh. Methods: A qualitative phenomenological approach was employed, involving semi-structured interviews with 25 participants aged 60 and above. Thematic analysis was utilized to analyze the data, allowing for the identification of key themes related to participants’ experiences with telehealth services. Results: Four main themes emerged from the analysis: access to technology and connectivity, attitudes toward telehealth, support systems, and institutional and policy factors. Participants reported challenges such as low digital literacy and unreliable Internet access, along with the need for trust in healthcare providers. Family support and the desire for training resources were highlighted as important facilitators of telehealth utilization. Conclusions: The findings indicate that addressing barriers such as digital literacy and connectivity is crucial for enhancing telehealth utilization among older adults. Implementing strategies that promote education, strengthen support systems, and improve policy frameworks is essential for facilitating greater engagement with telehealth services in this demographic.

## 1. Introduction

The aging population worldwide presents significant challenges for healthcare systems, necessitating innovative strategies to manage their complex needs [1]. According to the World Health Organization (WHO), the global population aged 60 years and older is expected to reach 2 billion by 2050, doubling from 1 billion in 2019 [2]. This demographic shift is accompanied by an increase in chronic diseases, higher healthcare utilization, and a pressing demand for healthcare resources [3]. In Saudi Arabia, the percentage of the population aged 60 and older is projected to significantly rise, leading to a greater need for accessible and effective healthcare services [4].

Older adults frequently encounter barriers to healthcare access, including mobility limitations, chronic health conditions, and the complexities of navigating traditional healthcare systems [5]. Telehealth has emerged as a promising solution to address these barriers by providing remote access to healthcare services, thus enhancing the convenience and efficiency of care delivery [6,7,8]. The COVID-19 pandemic accelerated the adoption of telehealth services, highlighting their potential to improve healthcare access for older adults, particularly during times of crisis [9,10]. However, the transition to telehealth has not been seamless, and several factors influence its acceptance and utilization among older populations [11,12].

The successful adoption of telehealth among older adults hinges on their technological proficiency, access to necessary devices, and Internet connectivity [13]. Many older individuals may lack the skills to effectively use digital platforms, which can lead to feelings of frustration and decreased willingness to engage with telehealth services [14]. Additionally, socioeconomic factors play a critical role, as older adults from lower-income backgrounds may not have access to the technology or Internet services required for telehealth [15,16].

Moreover, cultural attitudes toward healthcare technology significantly influence the willingness to adopt telehealth. In many cultures, including Saudi Arabia, there exists a preference for in-person consultations due to established norms and trust in face-to-face interactions [17]. This reluctance can be compounded by concerns regarding privacy and data security when using digital health platforms [18]. The implications of these cultural factors are critical, as they can hinder the effective implementation of telehealth initiatives targeting older adults [19,20].

Despite these challenges, telehealth services have demonstrated substantial benefits, including increased access to healthcare, improved management of chronic diseases, and enhanced patient engagement [21]. For older adults with chronic conditions, telehealth can facilitate regular monitoring and follow-up consultations, potentially reducing the need for hospital visits [22]. The integration of telehealth into routine care can enhance the quality of life for older adults, enabling them to manage their health conditions more effectively from the comfort of their homes [23,24].

In Saudi Arabia, the government has recognized the importance of telehealth in enhancing healthcare delivery, particularly for the aging population. Initiatives aimed at expanding telehealth services have been implemented, with a focus on improving accessibility and addressing the unique needs of older adults [25]. However, despite these efforts, the utilization rates of telehealth among older individuals remain low, indicating a gap between availability and acceptance [26].

Recent studies have demonstrated varying levels of telehealth adoption, influenced by factors such as age, socioeconomic status, geographic location, and technological accessibility. For example, older adults, while significantly benefiting from telehealth, often show lower adoption rates compared with younger populations, primarily due to challenges like reduced digital literacy and accessibility issues [27]. Geographic disparities also play a critical role, with rural areas experiencing lower adoption rates due to infrastructure challenges, sharply contrasting with urban settings where telehealth is more readily embraced [28].

Internationally, the scenario presents similarly complex dynamics. In countries like the United States and Canada, telehealth has seen a robust integration into healthcare systems, supported by substantial infrastructural investments [29]. Conversely, in less developed regions such as parts of Africa and South America, telehealth struggles with foundational challenges like reliable Internet access and healthcare provider training [30].

### 1.1. Theoretical Framework

To understand these diverse adoption patterns, several theoretical frameworks can be applied. The Technology Acceptance Model (TAM), developed by Davis (1989), is particularly relevant [31]. The TAM posits that two main factors influence the adoption of technology: perceived usefulness and perceived ease of use. In the context of telehealth, perceived usefulness might be influenced by the immediate benefits telehealth offers, such as reduced need for travel and enhanced access to specialists. Perceived ease of use, however, can be hindered by the complex interfaces or unreliable technology that older adults might face [32].

Additionally, sociological approaches to technology adoption also provide valuable insights, particularly when considering older adults. These approaches consider the broader social context influencing technology use, including social support networks, cultural attitudes toward technology, and the role of healthcare providers in facilitating technology adoption [33].

### 1.2. Aim of the Study

The aim of this study is twofold: (1) to explore the utilization of telehealth services among older adults in Saudi Arabia and (2) to identify the barriers and facilitators that influence their adoption and sustained use of these technologies in healthcare settings. Accordingly, our research questions are:

▪ What are the utilization patterns of telehealth services among older adults in Riyadh?
▪ What barriers and facilitators influence the adoption and sustained use of telehealth services among this demographic?

## 2. Materials and Methods

### 2.1. Study Design

This study employed a qualitative, phenomenological approach, utilizing Interpretative Phenomenological Analysis (IPA) as described by Smith et al. (2009) and J. A. Smith & Osborn (2015) [33,34]. The selection of IPA was motivated by the necessity to gain an in-depth understanding of the subjective experiences of older adults in utilizing telehealth services in Riyadh, Saudi Arabia. Phenomenology is particularly effective for exploring the lived experiences of individuals, making it an ideal method for examining how older adults perceive the barriers and facilitators impacting their adoption of telehealth technologies. The phenomenological approach is supported by existing research on telehealth adoption, which underscores the importance of understanding the personal and contextual factors that influence individuals’ acceptance and use of technology. For instance, studies by Zhang, et al. (2023) highlight that older adults’ decisions to adopt telehealth are deeply influenced by their personal experiences with technology, perceived usefulness, and ease of use—factors that are best explored through a phenomenological lens [35]. Furthermore, phenomenology allows researchers to uncover nuanced insights into the barriers and facilitators of telehealth adoption, such as emotional, psychological, and social factors, which might not be evident through other qualitative methods [36].

### 2.2. Participants and Setting

Participants for this study were older adults aged 60 and above residing in Riyadh City, Saudi Arabia. A purposive sampling strategy was employed to select individuals, ensuring a diverse representation in terms of sex, health status, and previous experience with telehealth services. This diversity allowed for a comprehensive understanding of the various perspectives on the challenges and supports affecting telehealth utilization. Participants were recruited from various community centers, healthcare facilities, and online platforms to gather insights reflecting different experiences and contexts related to telehealth services.

### 2.3. Inclusion and Exclusion Criteria

The inclusion criteria for participation were as follows:

- Older adults aged 60 years and above.
- Individuals who have previously used telehealth services or expressed interest in using them.
- Willingness to participate in this study and provide informed consent.

The exclusion criteria were as follows:

- Individuals who did not reside in Riyadh City.
- Those who had cognitive impairments affecting their ability to engage in this study.
- Individuals unwilling or unable to provide informed consent.

### 2.4. Data Collection

Participants were voluntarily recruited through community announcements and outreach in healthcare facilities, with interested individuals contacting the lead researcher via phone or email. Data collection was conducted through semi-structured, in-depth interviews led by the primary researcher, ensuring a consistent approach to gathering data. The interviews were conducted in private locations, such as community centers or participants’ homes, to guarantee comfort and confidentiality, lasting between 60 and 90 min.

A total of 25 interviews (Appendix A) were conducted from August to October 2024, with data collection continuing until saturation was achieved—indicated by the repetition of themes and no new insights emerging. With participants’ consent, all interviews were audio-recorded and subsequently transcribed verbatim. The interviews began with demographic questions to gather background information and establish rapport, followed by open-ended questions aimed at exploring participants’ experiences with telehealth, perceived barriers to its use, and factors that facilitated its adoption. To ensure ease of communication, all interviews were conducted in Arabic, with transcripts later translated into English by a bilingual expert, followed by a secondary review to verify the accuracy of translations.

### 2.5. Data Analysis

The data were analyzed using thematic analysis following Braun and Clarke’s (2006) guidelines, encompassing several stages [37]:

1. Familiarization: The researcher deeply engaged with the data by reading the transcripts multiple times and noting initial thoughts.
2. Generating Initial Codes: Systematic coding was performed across the dataset to identify significant statements related to telehealth utilization, perceived barriers, and enabling factors.
3. Searching for Themes: Initial codes were organized into potential themes, which were then reviewed and refined to accurately reflect the participants’ experiences.
4. Reviewing Themes: Identified themes were examined in relation to both the coded data and the dataset as a whole, leading to the development of a thematic map.
5. Defining and Naming Themes: Each theme was clearly articulated to ensure that the analysis coherently reflected the data.
6. Producing the Report: The final stage involved creating a detailed report that illustrated the themes using selected excerpts from the interviews, presenting a comprehensive narrative of the findings.

### 2.6. Ethical Considerations

Ethical approval for this study was obtained from the Institutional Review Board (IRB) at King Saud University (IRB: 24-951 on 1 August 2024). Participants received an information sheet and consent form detailing this study’s purpose, procedures, potential risks, and benefits. Confidentiality was upheld by anonymizing the interview transcripts and securely storing the data. Participants were informed of their right to withdraw from this study at any time without consequence. Audio recordings were deleted following transcription, and all transcribed data will be securely stored for five years before destruction.

### 2.7. Rigor and Reflexivity

To ensure rigor, this study adhered to the Consolidated Criteria for Reporting Qualitative Research (COREQ) guidelines [38]. The following measures were implemented to enhance credibility, dependability, confirmability, and transferability:

- Credibility: The primary researcher conducted all interviews, ensuring consistency in data collection. The interview guide was reviewed by qualitative research experts and pilot-tested to ensure its effectiveness. Member-checking was performed by summarizing key points during interviews and verifying them with participants.
- Dependability: The research process, including decisions and actions taken throughout this study, was thoroughly documented in an audit trail to ensure transparency and enable potential replication.
- Confirmability: Researchers maintained field notes and reflective diaries, documenting thoughts, decisions, and potential biases, ensuring that conclusions were grounded in data rather than personal assumptions.
- Transferability: Detailed descriptions of the research context, participants, and procedures were provided to enable readers to assess the applicability of findings to other settings. This study aimed to offer insights transferable to similar healthcare contexts by thoroughly describing the sample and the environment.
- Inter-Coder ReliabilityTwo: Researchers independently coded the data, and discrepancies were resolved through discussion to ensure reliability.
- Triangulation: Triangulation was achieved by cross-referencing interview findings with existing literature and participant feedback during member checking.

### 2.8. Inter-Coder Reliability

To ensure the rigor and reliability of the thematic analysis, inter-coder reliability was systematically established throughout the coding process. Initially, two independent researchers, both skilled in qualitative data analysis, were engaged in the coding of the transcripts. Each researcher independently reviewed a subset of the interviews to generate initial codes. Following this individual coding phase, the researchers convened to compare and discuss their findings, aiming to reach a consensus on the coding scheme.

Discrepancies in coding were addressed through discussion, with a senior researcher available to mediate unresolved conflicts, ensuring that the final themes were robust and reflective of the data. This collaborative and iterative process not only enhanced the validity of the coding but also ensured a comprehensive representation of the participants’ perspectives.

## 3. Results

### 3.1. Characteristics of Participants

This study included 25 older adult participants, aged between 60 and 82 years (average age: 68 years), providing a diverse range of experiences related to telehealth utilization in Riyadh, Saudi Arabia. The sex distribution was relatively balanced, with 52% (n = 13) of the participants identifying as women and 48% (n = 12) as men, reflecting the demographic composition of the older adult population in the region (Table 1).

Participants had varying levels of education: 40% held a secondary school diploma (n = 10), 32% had completed a bachelor’s degree (n = 8), and 28% had some post-secondary education or vocational training (n = 7). This educational diversity contributed to a wide range of perspectives regarding telehealth services and their accessibility.

The participants’ health status varied, with 48% (n = 12) reporting one or more chronic conditions, including diabetes, hypertension, and arthritis. Approximately 36% (n = 9) described themselves as having moderate health, while 16% (n = 4) reported good health. Prior experience with telehealth services was also diverse: 44% (n = 11) had utilized telehealth at least once, while the remaining 56% (n = 14) expressed interest in using such services but had not yet done so. This variety in age, sex, education, health status, and previous telehealth experience provided a comprehensive foundation for exploring the barriers and facilitators influencing telehealth utilization among older adults.

### 3.2. Thematic Analysis

The results of the thematic analysis of the interview data revealed four primary themes, each with distinct subthemes, illustrating the barriers and facilitators experienced by older adults in utilizing telehealth services in Riyadh, Saudi Arabia (Table 2). These themes encapsulate the complex experiences of participants, highlighting both systemic and individual factors that influence telehealth adoption.

#### 3.2.1. Access to Technology and Connectivity

##### Digital Literacy and Confidence

Participants expressed that varying levels of digital literacy significantly impacted their ability to effectively utilize telehealth services. Many older adults reported feeling overwhelmed by the technology, which hindered their willingness to engage with telehealth platforms. P5 shared, “I know how to use my phone for calls and messages, but when it comes to apps, I feel lost. It’s daunting”. Similarly, P12 noted, “I want to use telehealth, but I’m afraid I will mess something up. I need someone to show me how to do it”. These sentiments highlight how a lack of confidence in using digital tools can act as a barrier to telehealth engagement among older adults. This lack of digital literacy often stems from insufficient exposure to technology throughout their lives, leading to anxiety and reluctance to adopt new healthcare solutions. Many older adults reported feeling overwhelmed by the technology, which hindered their willingness to engage with telehealth platforms. Access to reliable Internet connectivity emerged as another critical factor influencing telehealth utilization.

##### Internet Connectivity and Access

Access to reliable Internet connectivity emerged as another critical factor influencing telehealth utilization. Participants living in areas with limited Internet services reported significant challenges in accessing telehealth consultations. P8 explained, “Sometimes the Internet is too slow, and I miss appointments because of connection issues”. P14 echoed this concern, stating, “I’d use telehealth more often if I didn’t have to worry about my Internet cutting out. It makes me anxious”. The concern over connectivity not only affects their ability to attend virtual consultations but also creates uncertainty about the effectiveness of telehealth as a viable healthcare option. This theme underscores the importance of stable Internet access as a facilitator for effective telehealth use among older adults, suggesting that improving infrastructure can enhance telehealth adoption rates. However, despite these technical challenges, many participants recognized the significant benefits of telehealth that can transform their access to healthcare services.

#### 3.2.2. Attitudes Toward Telehealth

##### Perceived Benefits of Telehealth

Many participants acknowledged the benefits of telehealth, particularly regarding convenience and accessibility. P10 remarked, “It’s nice to have a doctor’s appointment without having to leave my house. It saves me so much time and effort”. Additionally, P15 noted, “Telehealth is great for quick consultations; I don’t have to wait weeks for an in-person visit”. These positive perceptions reflect a general enthusiasm for telehealth services, especially among those with mobility issues or chronic conditions requiring frequent medical attention. Participants recognized that telehealth can facilitate timely interventions and reduce the physical and emotional toll associated with travel for medical appointments. Yet, for these advantages to be fully realized, trust in the quality of care provided via telehealth remains a crucial component that some participants felt was lacking. Despite acknowledging the benefits, participants expressed concerns about the quality of care received through telehealth.

##### Trust in Healthcare Providers

For many older adults, the interpersonal aspects of healthcare delivery are critical, and the perceived lack of personal connection during virtual consultations may deter them from fully embracing telehealth. Despite acknowledging the benefits, participants expressed concerns about the quality of care received through telehealth. Trust in healthcare providers was a significant factor in their willingness to engage with telehealth services. P3 stated, “I worry about whether the doctor can really help me if they can’t examine me in person”. P7 added, “I feel more comfortable seeing my doctor face-to-face. There’s a connection that’s lost online”. This theme highlights the importance of maintaining trust and connection in healthcare relationships, even when utilizing technology. For many older adults, the interpersonal aspects of healthcare delivery are critical, and the perceived lack of personal connection during virtual consultations may deter them from fully embracing telehealth. Addressing these concerns often requires the involvement of family and caregivers, who play an essential role in supporting older adults through the telehealth process. The role of family members and caregivers emerged as a crucial facilitator for telehealth utilization.

#### 3.2.3. Support Systems

##### Family and Caregiver Support

The role of family members and caregivers emerged as a crucial facilitator for telehealth utilization. Many participants reported relying on family members for assistance with technology and navigation of telehealth platforms. P6 shared, “My daughter helps me set up my appointments and reminds me how to use the app. Without her, I wouldn’t know where to start”. Similarly, P9 mentioned, “Having someone to guide me through the process makes it much less stressful”. This reliance on family support emphasizes the need for a collaborative approach to telehealth for older adults. Additionally, the presence of a supportive caregiver can enhance confidence in using telehealth services, reducing feelings of isolation and uncertainty about navigating the technology.

##### Availability of Resources and Training

Participants expressed a desire for more resources and training to enhance their understanding of telehealth services. Many suggested that community workshops or informational sessions would significantly benefit their ability to confidently use these technologies. P11 stated, “If there were classes for older adults on how to use telehealth, I would definitely attend”. This insight reflects a demand for structured educational opportunities that can empower older adults in their healthcare journey. Providing targeted training programs can improve digital literacy and increase overall comfort with telehealth, facilitating a smoother transition to this mode of healthcare delivery.

#### 3.2.4. Institutional and Policy Factors

##### Healthcare Provider Training

The adequacy of training provided to healthcare professionals regarding telehealth services was a concern among participants. Some expressed that providers may not be fully equipped to effectively facilitate telehealth consultations, impacting their overall experience. P4 noted, “Sometimes, I feel like the doctor doesn’t know how to use the system either, which makes me uncomfortable”. This observation highlights the need for ongoing training for healthcare providers to enhance their competency in telehealth delivery. Ensuring that providers are well trained not only improves the quality of care but also helps build patient confidence in using telehealth services.

##### Policy Support for Telehealth Services

Participants indicated that supportive policies promoting telehealth services are essential for increasing accessibility. Some expressed frustration with the bureaucracy surrounding telehealth adoption, suggesting that more streamlined processes would facilitate easier access to these services. P1 explained, “If it were easier to sign up for telehealth or if insurance covered it better, I think more people would use it”. This insight emphasizes the role of policy in shaping the availability and acceptance of telehealth services for older adults. The need for comprehensive policies that support the integration of telehealth into standard healthcare practices is crucial for enhancing utilization among the elderly population.

##### Barriers to Telehealth Adoption Among Non-Users

Despite the potential benefits of telehealth, more than half of our participants had never used these services. The primary reasons cited for not using telehealth include:

- Lack of Awareness: Several participants were unaware of the availability of telehealth services or did not fully understand how these services can be accessed or used to their benefit.
- Technological Challenges: Many of the non-users cited a lack of technical skills and access to the necessary technology—such as smartphones or computers—as major barriers.
- Cultural Preferences for In-Person Visits: A significant number of older adults expressed a strong preference for face-to-face interactions with healthcare providers, believing it leads to better care and more personal attention.
- Privacy Concerns: Concerns about data security and privacy were also mentioned, with participants unsure about the safety of sharing personal health information online.
- Perceived Irrelevance: Some felt that telehealth services were not suited to their specific health needs, which often include complex chronic conditions requiring physical examination.

## 4. Discussion

The discussion of this study highlights the multifaceted factors influencing the utilization of telehealth services among older adults in Riyadh, Saudi Arabia, with implications that extend to policy, practice, and future research. The findings revealed significant barriers and facilitators that shaped participants’ experiences with telehealth, underscoring themes such as digital literacy, Internet connectivity, trust in healthcare providers, family support, and policy frameworks.

Digital literacy emerged as a significant barrier among older adults, aligning with previous literature that emphasizes how technological proficiency affects telehealth adoption. Older adults often lack familiarity with digital tools, which creates anxiety and reluctance to engage in telehealth [39,40]. A study found that digital literacy among older populations is associated with the extent of exposure to technology during earlier life stages, influencing their confidence in using modern health platforms [41]. The current study’s participants echoed these sentiments, revealing that inadequate knowledge limited their ability to effectively utilize telehealth. Addressing this barrier requires targeted interventions that improve digital literacy through educational workshops and training programs, which have been shown to increase older adults’ comfort and competence with digital health solutions [42,43].

Internet connectivity was another notable challenge, as reliable access to digital infrastructure is essential for the effective use of telehealth. This issue is consistent with findings from other studies in the Middle East, where disparities in Internet access persist, especially among older and rural populations [44,45]. Inadequate Internet service can disrupt telehealth consultations, leading to frustration and decreased trust in the system’s reliability. The current findings resonate with the work of Zhang et al. (2022), who demonstrated that limited connectivity significantly impacts the continuity and quality of telehealth interactions [46]. Addressing this issue requires investments in digital infrastructure to ensure broader, stable Internet access, especially in regions with underserved populations [47].

The participants’ attitudes toward telehealth revealed mixed perspectives. While many acknowledged the convenience and time-saving benefits, there was a pervasive concern about the perceived quality of care remotely delivered. Trust in healthcare providers is a pivotal factor influencing telehealth utilization. The current study’s findings are supported by research conducted by Pogorzelska et al. (2022), which highlighted that the absence of physical interaction can hinder the development of trust, thereby impacting the perceived efficacy of telehealth consultations [48]. Maintaining trust necessitates strategies that enhance communication skills in virtual settings, ensuring that patients feel heard and understood despite the lack of face-to-face contact [49]. This aligns with the study by Almathami et al. (2020), which stressed that telehealth providers should receive training in building rapport through digital platforms to foster patient trust [50].

Family and caregiver support were critical facilitators that influenced telehealth engagement among participants. Many older adults relied on family members for assistance with navigating telehealth platforms and scheduling appointments. This reliance is corroborated by Graven et al. (2021), who found that family involvement can significantly boost telehealth use among older populations by providing practical help and emotional encouragement [51]. The role of caregivers as mediators in telehealth access highlights the importance of involving family members in telehealth education and training [52]. Moreover, policies should encourage caregiver engagement as an integral part of telehealth service delivery, ensuring that older adults receive the necessary support to optimize their healthcare experience [15].

Our findings reveal several key barriers to telehealth adoption among older adults in Riyadh, including digital literacy, technological accessibility, and cultural attitudes toward healthcare technology. These results are consistent with studies conducted in similar contexts, such as research in the United States, which identified digital literacy as a significant barrier among older populations [41]. However, our study extends these findings by highlighting specific cultural factors unique to Saudi Arabia, such as the preference for face-to-face medical interactions, which is less pronounced in Western contexts [8,17,53,54,55,56]

Unlike previous studies that have broadly addressed telehealth adoption barriers across various demographics, our research specifically pinpoints the nuances of these barriers in the context of Saudi older adults. For instance, Al-Sulimani’s (2023) findings suggest that while digital literacy is a common issue globally, the degree to which it influences telehealth adoption significantly varies with cultural and social norms regarding technology use among seniors [57]. Our study contributes to this discussion by offering detailed insights into how these norms manifest in Saudi Arabia, thus providing a targeted approach to addressing these barriers.

The detailed exploration of cultural attitudes in our study suggests a need for more tailored telehealth implementation strategies that consider local cultural dynamics. Future research can explore intervention strategies that specifically target cultural resistance to telehealth technology, potentially comparing the effectiveness of these strategies across different cultural settings. This approach not only provides a deeper understanding of the barriers but also opens avenues for developing more effective telehealth services that are culturally congruent and more readily accepted by older adults.

This study also underscored the need for resources and training aimed at older adults to bridge the digital divide. Participants expressed a strong interest in workshops and educational programs that would bolster their understanding and comfort with telehealth. This finding aligns with the research by Bahadori et al. (2024), which demonstrated that targeted training programs can significantly enhance digital competence among older adults, leading to increased adoption and sustained use of telehealth services [58]. Training programs not only build practical skills but also reduce the anxiety associated with using new technology, contributing to a more positive user experience [59]. Policymakers and healthcare organizations should prioritize the development of accessible, user-friendly educational initiatives to promote telehealth literacy [60].

Healthcare provider training was also highlighted as an area needing improvement. Participants noted that healthcare professionals’ lack of expertise in telehealth systems can negatively impact their experience. Continuous professional development programs focusing on telehealth competencies can improve the quality of virtual healthcare delivery, ensuring that both patients and providers are adequately equipped for effective communication [61].

Concerns over privacy and data security are paramount among telehealth users. To address these, we recommend the adoption of advanced encryption technologies and stringent data access controls. Additionally, continuous security training for healthcare providers should be mandated to ensure that they are equipped to securely handle sensitive patient information, maintaining the integrity of telehealth interactions [7].

Our study also explores cultural attitudes toward telehealth in Saudi Arabia, comparing these findings with global trends. Similar studies in Europe and Asia indicate shared concerns about telehealth, such as the impersonal nature of service and data privacy, suggesting that these barriers are not unique to Saudi Arabia but are instead globally observed. Understanding these universal barriers can help tailor telehealth systems to better meet the needs of diverse populations.

Policy support for telehealth emerged as an essential facilitator for increasing its adoption among older adults. Participants indicated that streamlined procedures and comprehensive policies are needed to make telehealth services more accessible and user-friendly. Simplified enrollment processes and policy-driven subsidies for telehealth services can remove barriers related to cost and accessibility [62]. Governments should also consider policies that enhance data security and privacy, as older adults often express concerns about the safety of their personal health information when using digital platforms [63].

The barriers to telehealth adoption identified among older adults in Riyadh resonate with global challenges, underscoring the universal need for strategic interventions to enhance digital healthcare accessibility. For instance, initiatives in regions like Scandinavia and Canada have demonstrated significant success in boosting telehealth usage among older populations [64]. In these countries, government-led programs focus on improving digital infrastructure, ensuring all demographics, especially the elderly, have reliable Internet access. Moreover, these regions have heavily invested in digital literacy programs specifically tailored for older adults, which include hands-on workshops and one-on-one training sessions provided in community centers and local libraries [65,66]. Such programs not only demystify technology but also make healthcare more accessible. Additionally, in response to cultural preferences for in-person visits, countries like the UK have introduced hybrid healthcare models, which combine traditional and telehealth services, allowing older adults to start with face-to-face appointments and gradually transition to remote consultations as they become more comfortable with the technology [19]. These examples can serve as a blueprint for Riyadh, suggesting that a combination of improved infrastructure, targeted educational initiatives, and flexible healthcare delivery models might overcome the current barriers and enhance telehealth adoption rates.

### 4.1. Implications of the Study

The findings of this study hold significant implications for healthcare policy, practice, and research related to telehealth services for older adults. Firstly, enhancing digital literacy among older populations is essential for increasing the utilization of telehealth services. Healthcare providers and policymakers should prioritize the development of educational programs that focus on building the digital skills of older adults, as well as providing resources for their caregivers. By addressing the knowledge gap and increasing confidence in using telehealth platforms, older adults may become more inclined to engage with these services, thus improving their overall healthcare access and outcomes.

To address the policy challenges identified, we propose specific actionable recommendations for policymakers. Firstly, we suggest the implementation of telehealth reimbursement policies that provide incentives for both healthcare providers and older adults, facilitating broader adoption. Secondly, targeted digital literacy programs designed for older adults should be established, enhancing their capabilities and comfort in using telehealth technologies, thus promoting wider utilization.

Additionally, the importance of fostering trust between older adults and healthcare providers cannot be overstated. Training programs for healthcare professionals should include modules on communication skills and relationship-building in virtual settings. By improving the quality of interactions during telehealth consultations, healthcare providers can enhance patient satisfaction and adherence to care recommendations.

To effectively enhance telehealth utilization among older adults in Riyadh, specific policy measures must be enacted. Firstly, subsidizing telehealth technologies and improving broadband infrastructure can ensure that all demographics, especially the elderly, have necessary access. Community-based training initiatives are also crucial; establishing local ‘telehealth hubs’ within community centers can provide hands-on training and access points for seniors. Moreover, incentivizing healthcare providers through tax breaks or subsidies for telehealth services can encourage adoption. Public awareness campaigns can further illuminate the benefits and availability of telehealth, helping to build trust and familiarity. Additionally, implementing strict regulatory frameworks to safeguard privacy and partnering with NGOs can tailor services to older adults’ needs, ensuring inclusivity and trust in telehealth systems. These combined efforts can significantly bolster telehealth’s adoption, making healthcare more accessible and tailored for Riyadh’s aging population.

### 4.2. Limitations of the Study

While this study provides valuable insights into the factors affecting telehealth utilization among older adults in Riyadh, it has several limitations that should be considered. Firstly, the qualitative nature of this study limits the generalizability of the findings. The experiences shared by participants may not represent the broader older adult population, particularly those living in different regions or with varying socioeconomic statuses.

Additionally, the sample size, while adequate for qualitative research, was relatively small and may not capture the full diversity of experiences among older adults. Future studies can benefit from larger and more diverse samples to validate and expand upon these findings.

Another limitation pertains to the reliance on self-reported data, which can introduce bias. Participants may have had difficulty accurately recalling their experiences or may have provided socially desirable responses. Triangulating data collection methods, such as incorporating observational studies or surveys, can enhance the reliability of the findings.

Finally, this study was conducted in a specific cultural context, which may influence the perceptions and experiences of telehealth among older adults. Cultural attitudes toward technology, healthcare, and family involvement can widely vary, and caution should be exercised when applying these findings to different cultural settings.

## 5. Conclusions

In conclusion, this study highlights the multifaceted barriers and facilitators influencing the utilization of telehealth services among older adults in Riyadh, Saudi Arabia. Key factors identified include digital literacy, Internet connectivity, trust in healthcare providers, family support, and the role of supportive policies. Addressing these challenges is critical to promoting telehealth as a viable healthcare option for older adults, particularly in the context of an aging population.

The findings emphasize the importance of comprehensive strategies that involve improving digital literacy, enhancing Internet access, and fostering trust between patients and providers. Furthermore, policy initiatives aimed at supporting telehealth adoption must prioritize the needs of older adults to ensure equitable healthcare access. This study contributes to the growing body of literature advocating for telehealth as a means to improve healthcare delivery for seniors, laying the groundwork for future research and policy development in this area.

## Figures and Tables

**Table 1:** Demographic Characteristics of Participants.

Characteristic	Category	Number of Participants	Percentage (%)
**Age**			
60–65 years		10	40%
66–75 years		9	36%
76–82 years		6	24%
**Sex**			
Women		13	52%
Men		12	48%
**Education Level**			
Secondary school diploma		10	40%
Bachelor’s degree		8	32%
Post-secondary/vocational	Associate degrees	4	16%
	Certificates	3	12%
**Health Status**			
Good		4	16%
Moderate		9	36%
Chronic conditions	Diabetes	5	20%
	Hypertension	4	16%
	Arthritis	3	12%

**Table 2:** Summary of Themes, Subthemes, and Participant Quotes.

Theme	Subtheme	Description	Example Quotes
**Access to Technology and Connectivity**			
	Digital Literacy and Confidence	Varying levels of digital literacy impact willingness to engage with telehealth; anxiety around technology affects use.	“I know how to use my phone for calls and messages, but when it comes to apps, I feel lost. It’s daunting.”—P5
	Internet Connectivity and Access	Reliable Internet access is crucial for effective telehealth utilization; connectivity issues can deter engagement.	“Sometimes the Internet is too slow, and I miss appointments because of connection issues.”—P8
**Attitudes Toward Telehealth**			
	Perceived Benefits of Telehealth	Participants recognize the convenience of telehealth for accessing healthcare; saves time and effort for those with mobility issues.	“It’s nice to have a doctor’s appointment without having to leave my house.”—P10
	Trust in Healthcare Providers	Concerns about the quality of care impact willingness to use telehealth services; maintaining a personal connection is crucial.	“I worry about whether the doctor can really help me if they can’t examine me in person.”—P3
**Support Systems**			
	Family and Caregiver Support	Family members play critical roles in both practical and emotional support for telehealth engagement.	
	Practical Support	Family helps with technology setup and navigation of telehealth platforms.	“My daughter helps me set up my appointments and reminds me how to use the app.”—P6
	Emotional Support	Emotional reassurance from family members encourages use of telehealth services.	“Having someone to guide me through the process makes it much less stressful.”—P9
**Institutional and Policy Factors**			
	Healthcare Provider Training	Adequate training for healthcare providers is necessary for effective telehealth; unprepared providers can impact patient experiences.	“Sometimes, I feel like the doctor doesn’t know how to use the system either, which makes me uncomfortable.”—P4
	Policy Support for Telehealth Services	Supportive policies can enhance accessibility and utilization of telehealth services; streamlined processes are needed to facilitate access.	“If it were easier to sign up for telehealth or if insurance covered it better, I think more people would use it.”—P1

## Data Availability

All data are available within the manuscript.

## Appendix A. Interview Questions

Research Question 1: What are the utilization patterns of telehealth services among older adults in Riyadh?
Interview Questions:

1. How frequently have you used telehealth services in the past year?
  ◦ This question aims to gauge the frequency of telehealth usage among older adults, directly addressing the study’s aim to explore utilization patterns.
2. What types of telehealth services have you accessed?
  ◦ This question helps identify the range of telehealth services utilized, further detailing the utilization patterns.

Research Question 2: What barriers and facilitators influence the adoption and sustained use of telehealth services among this demographic?
Interview Questions:

1. What challenges have you faced while using telehealth services?
  ◦ Designed to uncover any barriers that older adults might encounter, which hinders the adoption of telehealth.
2. What features of telehealth services do you find helpful?
  ◦ This question explores the facilitators that encourage the continued use of telehealth services.
3. What improvements would encourage you to use telehealth services more often?
  ◦ Targets potential facilitators by identifying changes that could make telehealth more attractive for sustained use.
4. How do family members or caregivers support your use of telehealth services?
  ◦ Investigates the role of family and caregivers as facilitators in the adoption and sustained use of telehealth.
